# Powdered Green Tea (Matcha) Attenuates the Cognitive Dysfunction via the Regulation of Systemic Inflammation in Chronic PM_2.5_-Exposed BALB/c Mice

**DOI:** 10.3390/antiox10121932

**Published:** 2021-11-30

**Authors:** Jong Min Kim, Jin Yong Kang, Seon Kyeong Park, Jong Hyun Moon, Min Ji Kim, Hyo Lim Lee, Hye Rin Jeong, Jong Cheol Kim, Ho Jin Heo

**Affiliations:** 1 Division of Applied Life Science (BK21), Institute of Agriculture and Life Science, Gyeongsang National University, Jinju 52828, Korea; myrock201@gnu.ac.kr (J.M.K.); kangjy2132@wikim.re.kr (J.Y.K.); skp210316@kbri.com (S.K.P.); 2020210043@gnu.ac.kr (J.H.M.); minjee9790@gnu.ac.kr (M.J.K.); gyfla059@gnu.ac.kr (H.L.L.); gpfls1428@gnu.ac.kr (H.R.J.); 2 Advanced Process Technology and Fermentation Research Group, World Institute of Kimchi, Gwangju 61755, Korea; 3 Department of Neural Development and Disease, Korea Brain Research Institute (KBRI), Daegu 41062, Korea; 4 Institute of Hadong Green Tea, Hadong 52304, Korea; jckim@hgreent.or.kr

**Keywords:** powdered green tea, matcha, particulate matter, PM_2.5_, systemic inflammation, olfactory bulb, hippocampus

## Abstract

This study was conducted to evaluate the anti-amnesic effect of the aqueous extract of powdered green tea (matcha) (EM) in particulate matter (PM)_2.5_-induced systemic inflammation in BALB/c mice. EM ameliorated spatial learning and memory function, short-term memory function, and long-term learning and memory function in PM_2.5_-induced mice. EM protected against antioxidant deficit in pulmonary, dermal, and cerebral tissues. In addition, EM improved the cholinergic system through the regulation of acetylcholine (ACh) levels and acetylcholinesterase (AChE) activity in brain tissue, and it protected mitochondrial dysfunction by regulating the production of reactive oxygen species (ROS), mitochondrial membrane potential (MMP) and ATP contents in brain tissue. EM attenuated systemic inflammation and apoptotic signaling in pulmonary, dermal, olfactory bulb, and hippocampal tissues. Moreover, EM suppressed neuronal cytotoxicity and cholinergic dysfunction in hippocampal tissue. This study suggests that EM might be a potential substance to improve PM_2.5_-induced cognitive dysfunction via the regulation of systemic inflammation.

## 1. Introduction

Air pollution is a complex mixture of particulate matter (PM) that contains heavy metals, carbon monoxide, sulfur dioxide, polycyclic aromatic hydrocarbons (PAHs), and ozone, and is known to be harmful to human health [1]. PM, which is classified as PM_10_ (≤10 µM) and PM_2.5_ (≤2.5 µM), occurs due to the rapid development of industries around the world. In particular, PM_2.5_ is commonly considered an indicator of air pollution and exhibits detrimental toxicity in the human body [2]. PM_2.5_ is absorbed through a variety of pathways, such as skin, nasal cavity, respiratory organs, and digestive system, and causes type 2 diabetes, respiratory infections, cardiovascular disease, and systemic inflammation. Most studies on PM_2.5_ are related to respiratory and cardiovascular damage, but the possibility of affecting various tissues is increasing [1,3,4]. PM_2.5_ promotes pulmonary and dermal inflammatory responses and causes systemic inflammation through the circulatory system. It causes the secretion of cytokines, such as tumor necrosis factor-α (TNF-α), interleukin-1β (IL-1β), and interleukin-6 (IL-6) in the serum, which reach brain tissue [5]. In particular, PM_2.5_ passes directly through the nasal cavity due to its small size and causes blood–brain barrier (BBB) disruption and damage to olfactory bulb and hippocampal tissues related to pathological dysfunction of the CNS [1]. PM_2.5_ alters synaptic gene expression, causing neurological disorders, and ultimately causes early behavior and Alzheimer’s disease (AD)-like neuropathology [6].

Matcha, the powdered form of green tea (*Camellia sinensis*), is known to contain a variety of physiological and bioactive compounds, such as tannins, phenolic acids, and catechins [7]. In particular, large amounts of catechins, such as (−)-epicatechin (EC), (−)-epigallocatechin (EGC), (−)-epicatechin-3-gallate (ECG), and (−)-epigallocatechin-3-gallate (EGCG), are included in matcha as opposed to other processed green tea, such as hojicha, as well as oolong tea and black tea [8]. Matcha catechins present various physiological activities, such as anti-inflammatory effects, renal and neuroprotective effects, cholesterol accumulation inhibitory effects, and anti-diabetic effects, which are derived from the structural features of flavan-3-ol [9,10,11]. In addition, matcha also contains many phenolic acids, such as chlorogenic acid, gallic acid, quercetin, and kaempferol, which have anti-amnesic effects, non-alcoholic fatty liver disease inhibitory effects, and antibacterial effects [12,13]. Furthermore, in a previous study, matcha showed significantly higher catechin contents and antioxidant capacities than leaf green tea (Tables S1 and S2) [14]. As various studies have reported its physiological activity, the evaluation of the health benefits of matcha is continuously required.

However, there are few studies related to the protective effect of matcha against cognitive impairment caused by PM_2.5_ exposure. Thus, the protective effect of matcha was analyzed by evaluating the in vivo behavior, cognitive function, and regulatory effects on systemic inflammation in PM_2.5_-induced BALB/c mice.

## 2. Materials and Methods

### 2.1. Chemicals

Dimethyl sulfoxide, hydroxylamine·hydrochloride, phenylmethane sulfonylfluoride, thiobarbituric acid, metaphosphoric acid, o-phthaldialdehyde, bovine serum albumin, HEPES, digitonin, 2′,7′-dichlorofluorescein diacetate (DCF-DA), tetrachloro-1,1,3,3-tetraethylbenzimidazolylcarbo-cyanine iodide (JC-1), iron (III) chloride hexahydrate, egtazic acid (EGTA), malate, pyruvate, phosphoric acid, metaphosphoric acid, protease inhibitor, polyvinylidene difluoride (PVDF) membrane, Dulbecco’s modified Eagle’s medium (DMEM), fetal bovine serum, penicillin, streptomycin, 2′,3-(4,5-dimethylthiazol-2-yl)-2,5-diphenyl tetrazolium bromide (MTT), and solvents were purchased from Millipore (Billerica, MA, USA). A superoxide dismutase (SOD) determination kit was purchased from Dojindo Molecular Technologies (Kumamoto, Japan). An ATP bioluminescence assay kit was purchased from Promega Corp. (Madison, WI, USA). PM_2.5_ (mean diameter: 1.06 μm) was purchased from Power Technology INC. (Arizona Test Dust (ATD), Arden Hills, MN, USA). The components of PM_2.5_ are presented in Table 1.

### 2.2. Sample Preparation

Matcha was provided by the Institute of Hadong Green Tea (Hadong, Korea). Shade cultured green tea was cultivated for about 3 weeks to block the UV so as to improve the palatability. The cultivated green tea was superheated at more than 250 °C for 1 min and rapidly cooled (900K-1; Kawasaki Tea Machinery, Kakegawa, Japan). The cooled green tea was dried and powdered using a bead mill with a millstone (rotational speed of 50 to 55 rpm; particle size of 13 to 14 μm) (Kawasaki Tea Machinery) to produce the matcha. The matcha was lyophilized using a freeze drier (Operon, Gimpo, Korea) at −80 °C, and extracted with 50-fold distilled water at 40 °C for 2 h. The extracts were evaporated using a vacuum rotary evaporator (N-N series, Eyela Co., Tokyo, Japan), and re-lyophilized. The lyophilized extract of matcha (EM) was kept at −20 °C until use.

### 2.3. Animals and In Vivo Experimental Design

Six-week-old male BALB/c mice were obtained from Samtako (Osan, Korea). The experimental mice were randomly divided into four per cage and controlled in standard laboratory conditions with a 12 h light/dark cycle, 55% humidity, and 22 ± 2 °C. Experimental animal groups were divided into 6 groups (*n* = 20; 7 for in vivo tests; 5 for ex vivo tests; 5 for mitochondrial tests; 3 for Western blot analysis) as a sham control (Sham) group without chamber exposure, a clean air-exposed normal control (NC) group, a clean air-exposed control with EM treatment group (40 mg/kg of body weight; NS), a PM_2.5_-exposed group (negative control group; PM), and two PM_2.5_-exposed with EM treatment groups (20 and 40 mg/kg of body weight; EM20 and EM40, respectively). The EM was dissolved in drinking water and was orally fed using a stomach tube as a Zonde needle once a day for 12 weeks. The WHO air quality guidelines for fine dust recommend mean exposures of up to PM_2.5_ < 25 μg/m^3^ per 24 h for humans, and the annual PM_2.5_ concentration in the most polluted cities in the world is around 170 μg/m^3^ [15]. The mice were exposed to PM_2.5_ at a 500 μg/m^3^ concentration in the whole-body exposure chamber for 5 h per day for 12 weeks. The PM_2.5_ was dissolved in purified water and sprayed as an aerosol form (flow rate of 10 MFC; air speed of 10 L/min) by controlling an internal programmable logic controller. All animal experimental protocols were conducted according to the Institutional Animal Care and Use Committee of Gyeongsang National University (certificate: GNU-200302-M0007, approved on 2 March 2021) and performed in accordance with the Policy of the Ethical Committee of Ministry of Health and Welfare, Republic of Korea.

### 2.4. Behavioral Test

#### 2.4.1. Y-Maze Test

The dimensions of the Y-maze were 33 (length) × 15 (height) × 10 (width) cm. The mice were located at the end of a designated arm and allowed to freely move within the maze for 8 min [16]. The mice’s movements were recorded by a video tracking system (Smart 3.0, Panlab, Barcelona, Spain).

#### 2.4.2. Passive Avoidance Test

The passive avoidance acryl chamber was divided into an illuminated and non-illuminated part that could give electrical stimulation separated by a door. On the first day, the mice were located in the light cubicle. When all four feet of the mice entered the dark cubicle, a foot shock was applied at 0.5 mA for 3 s, and the first latency time was measured. In the test trial, the step-through latency to re-enter the electronic zone was measured [17].

#### 2.4.3. Morris Water Maze Test

The Morris water maze (MWM) pool consisted of a circular steel pool (90 cm in diameter and 30 cm deep) and was randomly divided into four zones marked as N, S, E, and W. The submerged platform was located in the center of the W quadrant. The mice were allowed to swim freely, and the mice’s movements were recorded using a video tracking system (Smart 3.0, Panlab). In the hidden trial test, each mouse swam and escaped over 4 days. Lastly, a probe trial was conducted without the platform, and the retention time was determined [18].

### 2.5. Preparation of Tissue

After an in vivo test, pulmonary, dorsal dermal, and cerebral tissues of the sacrificed mice were homogenized using a bullet blender (Next Advance Inc., Averill Park, NY, USA) with phosphate-buffered saline (PBS, pH 7.4) for acetylcholine (ACh), acetylcholinesterase (AChE), SOD, and malondialdehyde (MDA) contents, and phosphate buffer (pH 6.0) for reduced glutathione (GSH) contents. The protein concentration was measured using the Bradford protein assay [19].

### 2.6. Antioxidant System

#### 2.6.1. Ferric-Reducing/Antioxidant Power (FRAP) in Serum

Blood samples were obtained from the postcaval vein and collected in a heparin tube. The obtained samples were centrifuged at 10,000× *g* for 10 min at 4 °C and the plasma ferric-reducing/antioxidant power (FRAP) was measured. Sample and TPTZ solution, including 300 mM sodium acetate buffer (pH 3.6), 10 mM TPTZ in 40 mM HCl, and 20 mM FeCl_2_ were reacted for 15 min at 37 °C, and the absorbance of the reactant was measured at 593 nm using a microplate reader (Epoch 2, BioTek Instruments Inc., Winooski, VT, USA) [20].

#### 2.6.2. SOD Contents

To measure the SOD contents, the homogenized tissue in PBS was centrifuged at 400× *g* for 10 min at 4 °C to remove the components affecting SOD activity, such as vitamin C, reduced GSH, blood clots, and debris, and the pellets were used for SOD analysis. The pellets in 1 × cell extraction buffer (10% SOD buffer, 0.4% (*v/v*) Triton X-100, and 200 μM phenylmethane sulfonylfluoride) were centrifuged at 10,000× *g* for 10 min at 4 °C. The SOD contents were measured using a commercial SOD kit (Dojindo Molecular Technologies).

#### 2.6.3. Reduced GSH Contents

To measure the GSH contents, the homogenate at phosphate buffer (pH 6.0) was centrifuged at 10,000× *g* for 15 min at 4 °C. This supernatant was reacted with 5% metaphosphoric acid and re-centrifuged at 2000× *g*. The obtained supernatant was added to 0.26 M of tris-HCl (pH 7.8), 0.65 N of NaOH, and 1 mg/mL of o-phthaldialdehyde at room temperature for 15 min. The fluorescence was measured using a fluorescence microplate reader (Infinite 200, Tecan Co., Männedorf, Switzerland) at 320 nm (excitation) and 420 nm (emission) [21].

#### 2.6.4. MDA Contents

To measure the MDA contents, the homogenized tissues in PBS were centrifuged at 5000 rpm for 10 min at 4 °C. The supernatants were reacted with 1% phosphoric acid and 0.67% thiobarbituric acid in a 95 °C water bath for 1 h. The reactants were centrifuged at 600× *g* for 10 min, and the supernatants were measured at 532 nm [22].

### 2.7. Cerebral Cholinergic System

#### 2.7.1. ACh Contents

To measure the ACh contents, the supernatant was added to alkaline hydroxylamine reagent (2 M hydroxylamine·hydrochloride and 3.5 N sodium hydroxide, 1:1 (*v/v*)) and mixed at room temperature for 1 min. This mixture was mixed with 0.5 N of hydrochloride and 0.3 M of iron (III) chloride hexahydrate. The absorbance of the mixture was measured at 540 nm using a microplate reader (Epoch 2, BioTek Instruments Inc., Winooski, VT, USA) [23].

#### 2.7.2. AChE Activities

To measure the AChE activity, the supernatant was reacted with 50 mM of sodium phosphate buffer (pH 7.4) at 37 °C for 15 min This mixture was reacted with Ellman’s reaction mixture at 37 °C for 10 min. The absorbance of the mixture was measured at 405 nm using a microplate reader (Epoch 2, BioTek Instruments Inc.) [24].

### 2.8. Mitochondrial Activity

#### 2.8.1. Mitochondrial Isolation

The tissues were homogenized with 10-fold volumes of mitochondrial isolation (MI) buffer, including 215 mM of mannitol, 75 mM of sucrose, 0.1% bovine serum albumin, 20 mM of HEPES sodium salt (pH 7.2)] with 1 mM of EGTA, and centrifuged at 1300× *g* for 10 min at 4 °C to extract the mitochondria from the brain tissue. The supernatant was re-centrifuged again at 13,000× *g* for 10 min at 4 °C. The mitochondrial pellets were reacted with MI buffer and 0.1% digitonin for 5 min and the mixtures were reacted with 2 mL of MI buffer, including 1 mM of EGTA. The mixture was centrifuged at 13,000× *g* for 15 min at 4 °C, and this supernatant, as isolated mitochondria, was used for mitochondrial analysis.

#### 2.8.2. Mitochondrial ROS Contents

The mitochondrial reactive oxygen species (ROS) content was evaluated using the isolated mitochondrial sample. This mitochondrial sample was mixed with KCl-based respiration buffer comprised of 125 mM of potassium chloride, 2 mM of potassium phosphate monobasic, 20 mM of HEPES, 1 mM of MgCl_2_, 500 µM of EGTA, 2.5 mM of malate, 5 mM of pyruvate, and 25 μM of DCF-DA for 20 min. The fluorescence of the reactant was measured using a fluorescence microplate reader (Infinite 200, Tecan Co.) at 485 nm (excitation) and 530 nm (emission) [25].

#### 2.8.3. Mitochondrial Membrane Potential

The mitochondrial membrane potential (MMP) was measured using the isolated mitochondria. An MI buffer with 5 mM of pyruvate, 5 mM of malate, and the isolated mitochondria was gently reacted with 1 μM of JC-1 in a black 96-well plate. That was incubated at room temperature for 20 min, and then the fluorescence was measured using a fluorescence microplate reader (Infinite 200, Tecan Co.) at 530 nm (excitation) and 590 nm (emission) [25].

#### 2.8.4. ATP Contents

The ATP contents were measured using a commercial ATP bioluminescence assay kit (Promega Corp.) according to the manufacturer’s protocol. The ATP content was assessed using a luminescence meter (GloMax-Multi+, Promega) and evaluated according to a standard curve.

### 2.9. Western Blot

The pulmonary, dermal, olfactory bulb, and hippocampus tissues were homogenized for 10 min in lysis buffer (GeneAll Biotechnology, Seoul, Korea) with a 1% protease inhibitor. Protein levels were measured using obtained supernatant centrifuged at 13,000× g for 10 min at 4 °C were measured for protein analysis. The protein, which is relatively concentrated, was separated by the SDS-PAGE gel and electro-transferred to the PVDF membrane. The membranes were reacted overnight in primary antibodies at 4 °C, and secondary antibodies for 1 h at room temperature. The luminescence of the immune complexes was detected using a Western blot image analyzer (iBright Imager, Thermo-Fisher Scientific, Waltham, MA, USA). The protein densities were calculated by ImageJ software (National Institutes of Health, Bethesda, MD, USA). Antibody details are presented in Table 2.

### 2.10. Statistical Analysis

All results are presented as the mean ± standard deviation. Statistically significant differences among the groups were analyzed by one-way analysis and determined using Duncan’s new multiple-range test (*p* < 0.05) of SAS ver. 9.4 (SAS Institute Inc., Cary, NC, USA). The data were statistically represented as significantly different from the NC group (*) and significantly different from the PM group (^#^), respectively (* and ^#^ *p* < 0.05, ** and ^##^ *p* < 0.01).

## 3. Results

### 3.1. Behavioral Tests

#### 3.1.1. Y-Maze Test

**Figure 1:**
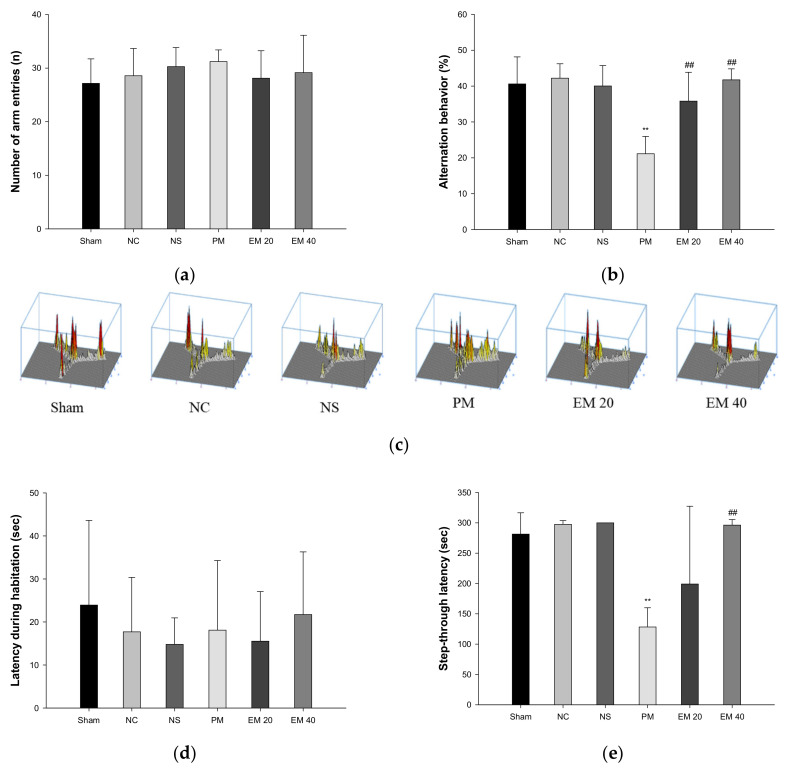
Protective effect of aqueous extract of matcha (EM) on the number of arm entries (**a**), alternation behavior (**b**), path tracing in Y-maze test (**c**), latency during habituation (**d**), and step-through latency in passive avoidance test (**e**). Results shown are mean ± SD (*n* = 7). Data were considered statistically significant at *p* < 0.05. Data are statistically represented with * = significantly different from the NC group, and ^#^ = significantly different from PM group; * and ^#^ *p* < 0.05, ** and ^##^ *p* < 0.01.

Spatial learning and memory function were estimated using a Y-maze test (Figure 1a–c). There were no significant differences in the number of arm entries among all the groups (Figure 1a). In the results of the alternation behavior tests, the Sham (40.61%), NC (42.20%), and NS (40.02%) groups showed no significant differences (Figure 1b). The results of the PM group (21.13%) were significantly more reduced than the NC group. On the other hand, the alternation behaviors of the EM groups (EM20, 35.82%; EM40, 41.73%) increased more than for the PM group (Figure 1b). In addition, as the concentration of EM increased, it was seen that spatial learning and memory function were considerably improved (Figure 1c).

#### 3.1.2. Passive Avoidance Test

Short-term learning and memory ability were measured using the passive avoidance test (Figure 1d,e). The first day of step-through latency showed no significant difference (Figure 1d). The step-through latencies on the second day for the Sham (281.41 s), NC (297.50 s), and NS (300.00 s) groups also showed no significant changes. Those of the PM group (128.29 s) were significantly more decreased than in the NC group. By contrast, those of the EM groups (EM20, 199.12 s; EM40, 296.45 s) increased more than those of the PM group (Figure 1e). Those of the EM 40 group were especially significantly improved.

#### 3.1.3. MWM Test

**Figure 2:**
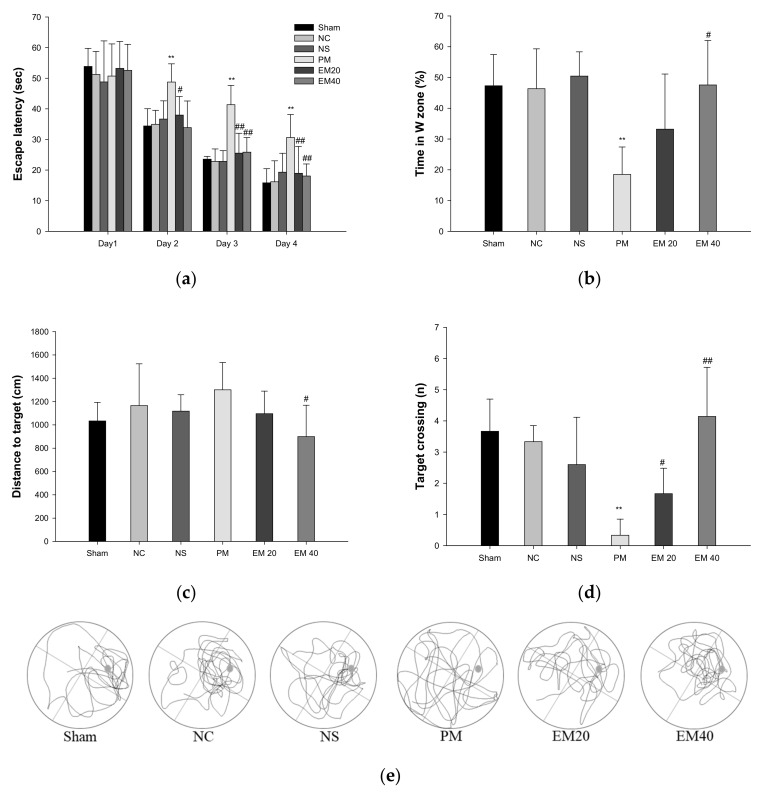
Protective effect of aqueous extract of matcha (EM) on escape latency in hidden trial (**a**), time in W zone (**b**), distance to target (**c**), target crossing (**d**), and path tracing (**e**) in the probe trial. Results shown are mean ± SD (*n* = 7). Data were statistically considered at *p* < 0.05, and data are statistically represented with * = significantly different from the NC group, and ^#^ = significantly different from PM group; * and ^#^ *p* < 0.05, ** and ^##^ *p* < 0.01.

Long-term learning and memory ability were investigated using an MWM test (Figure 2). In the results of the MWM test, there were no significant differences among the Sham, NC, and NS groups (Figure 2a). In the hidden test trial on the last training day, the escape latency of the PM group (30.64 s) decreased relatively less than the NC group (16.19 s). However, the escape latencies of the EM groups (18.95 s and 18.06 s) significantly more decreased than that for the PM group. In the probe trial, the relative retention time, distance to the target, and the target crossing in the W zone of the PM group (18.51%, 1361.03 cm, and 0.33) were significantly more reduced than those of the NC group (46.37%, 965.53 cm, and 3.33). On the other hand, the EM groups (EM20, 33.21%, 1168.03 cm; EM40, 1.67; 47.56%, 968.70 cm, and 4.14, respectively) showed an increased distance to the target and the target crossing compared to the PM group (Figure 2b–d). In comparing the tracked movements, long-term learning and memory ability were considerably more improved in the EM groups than in the PM group (Figure 2e).

### 3.2. Antioxidant System

#### 3.2.1. FRAP

**Figure 3:**
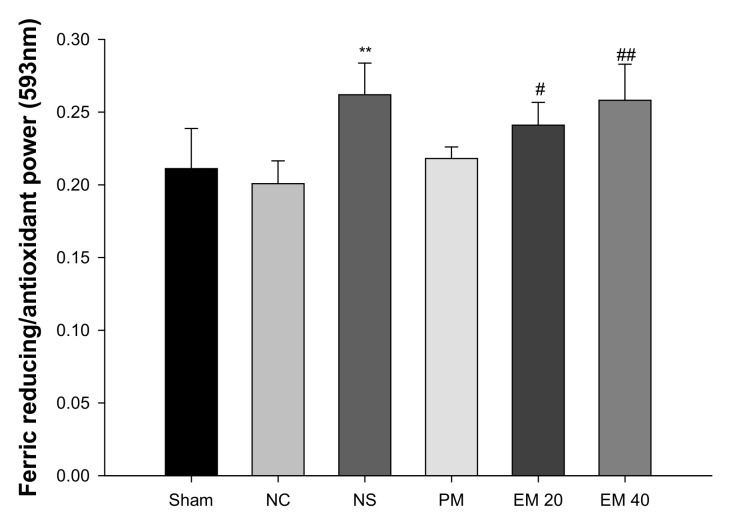
Protective effect of aqueous extract of matcha (EM) on ferric-reducing/antioxidant power (FRAP) in serum. Results shown are mean ± SD (*n* = 5). Data were statistically considered at *p* < 0.05, data are statistically represented with * = significantly different from the NC group, and ^#^ = significantly different from PM group; * and ^#^ *p* < 0.05, ** and ^##^ *p* < 0.01.

A FRAP assay was conducted to measure the antioxidant activity in serum (Figure 3). There were no significant differences in antioxidant power among the Sham group (0.21), NC group (0.20), and PM group (0.22). However, that of the NS group (0.26) and the EM groups (EM20, 0.24; EM40, 0.26) in serum were considerably increased by administrating the EM.

#### 3.2.2. SOD Activity

**Figure 4:**
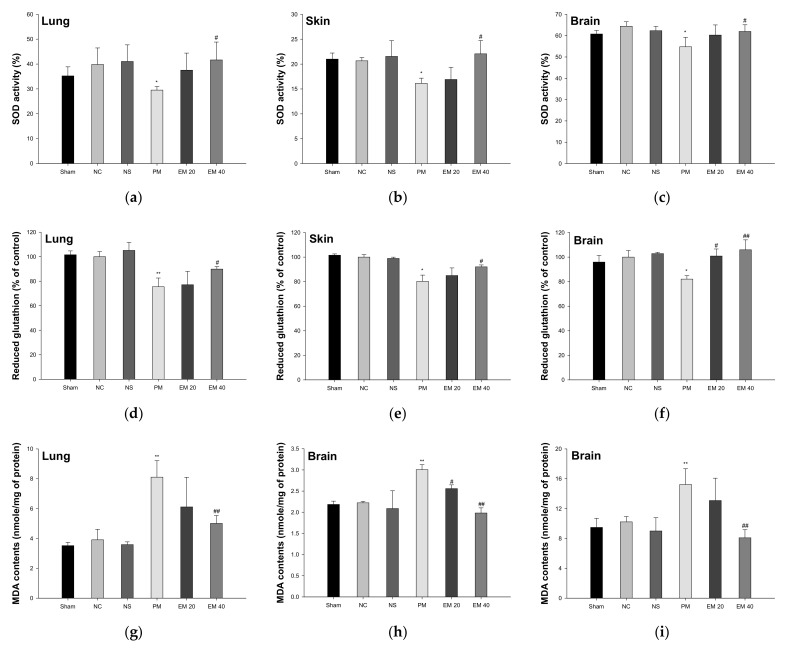
Protective effect of aqueous extract of matcha (EM) on superoxide dismutase (SOD) activity (**a**–**c**), reduced glutathione (GSH) contents (**d**–**f**) and malondialdehyde (MDA) contents (**g**–**i**) in pulmonary, dermal, and cerebral tissues. Results shown are mean ± SD (*n* = 5). Data were statistically considered at *p* < 0.05, and data are statistically represented with * = significantly different from the NC group, and ^#^ = significantly different from PM group; * and ^#^ *p* < 0.05, ** and ^##^ *p* < 0.01.

The SOD activity in the lung, skin, and brain tissues is shown in Figure 4a–c. The SOD activity in lung and brain tissue among the Sham (35.24%, 21.00%, and 60.79%, respectively), NC (39.82%, 20.65%, and 64.45%, respectively), and NS (41.04%, 21.54%, and 62.33%, respectively) groups showed no significant differences. That of the PM group (29.52%, 16.14%, and 54.79%, respectively) was significantly more reduced than in the NC group. However, that of the EM groups (EM20, 37.53%, 16.91%, and 60.79%, respectively; EM40, 41.65%, 22.07%, and 61.96%, respectively) in the lung and brain tissues was significantly more increased than in the PM group.

#### 3.2.3. Reduced GSH Contents

The reduced GSH contents in the lung, skin, and brain tissues are shown in Figure 4d–f. Reduced GSH contents in the lung and brain tissues among the Sham (101.55%, 101.57%, and 95.98% of the control, respectively), NC (100.00%, 100.00%, and 100.00% of the control, respectively), and NS (105.12%, 98.91%, and 102.85% of the control, respectively) groups showed no significant differences. Those of the PM group (75.51%, 80.25%, and 82.09% of the control, respectively) were significantly more reduced than the NC group. However, those of the EM groups (EM20, 77.09%, 85.01%, and 100.88% of the control, respectively; EM40, 89.86%, 92.09%, and 105.97% of the control, respectively) in the lung and brain tissues were significantly more increased than in the PM group.

#### 3.2.4. MDA Contents

The MDA contents in the lung, skin, and brain tissues are shown in Figure 4g–i. The MDA contents in the lung and brain tissues among the Sham (3.52 mmole/mg, 2.18 mmole/mg, and 9.48 mmole/mg of protein, respectively), NC (3.91 mmole/mg, 2.22 mmole/mg, and 10.22 mmole/mg of protein, respectively) and NS (3.58 mmole/mg, 2.09 mmole/mg, and 9.00 mmole/mg of protein, respectively) groups showed no significant differences. Those of the PM group (8.11 mmole/mg, 3.01 mmole/mg, and 15.23 mmole/mg of protein, respectively) were significantly more reduced than in the NC group. However, those of the EM groups (EM20, 6.11 mmole/mg, 2.56 mmole/mg, and 13.08 mmole/mg of protein, respectively; EM40, 5.01 mmole/mg, 1.98 mmole/mg, and 8.09 mmole/mg of protein, respectively) in the lung and brain tissues were significantly more attenuated than in the PM group.

### 3.3. Cholinergic System

#### 3.3.1. ACh Contents

**Figure 5:**
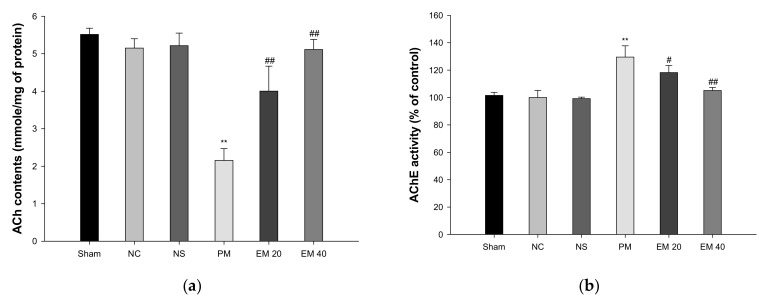
Protective effect of aqueous extract of matcha (EM) on acetylcholine (ACh) contents (**a**) and acetylcholinesterase (AChE) activity (**b**) in cerebral tissue. Results shown are mean ± SD (*n* = 5). Data were statistically considered at *p* < 0.05, and data are statistically represented with * = significantly different from the NC group, and ^#^ = significantly different from PM group; * and ^#^ *p* < 0.05, ** and ^##^ *p* < 0.01.

The cerebral ACh contents are shown in Figure 5a. The ACh contents of the Sham (5.52 mmole/mg of protein), NC (5.15 mmole/mg of protein), and NS (5.22 mmole/mg of protein) groups showed no significant differences. The ACh contents of the PM group (2.15 mmole/mg of protein) were significantly more reduced than those of the NC group. By contrast, those of the EM group (EM20, 4.00 mmole/mg of protein; EM40, 5.11 mmole/mg of protein) were significantly more restored than in the PM group.

#### 3.3.2. AChE Activities

The cerebral AChE activity is shown in Figure 5b. The AChE activity of the Sham (101.55%), NC (100.00%), and NS (99.20%) groups showed no significant differences. The AChE activity of the PM group (129.55%) was significantly more increased than that of the NC group. On the other hand, those of the EM group (EM20, 118.22%; EM40, 105.12%) were considerably more suppressed than the PM group.

### 3.4. Mitochondrial Activity

#### 3.4.1. Mitochondrial ROS Contents

**Figure 6:**
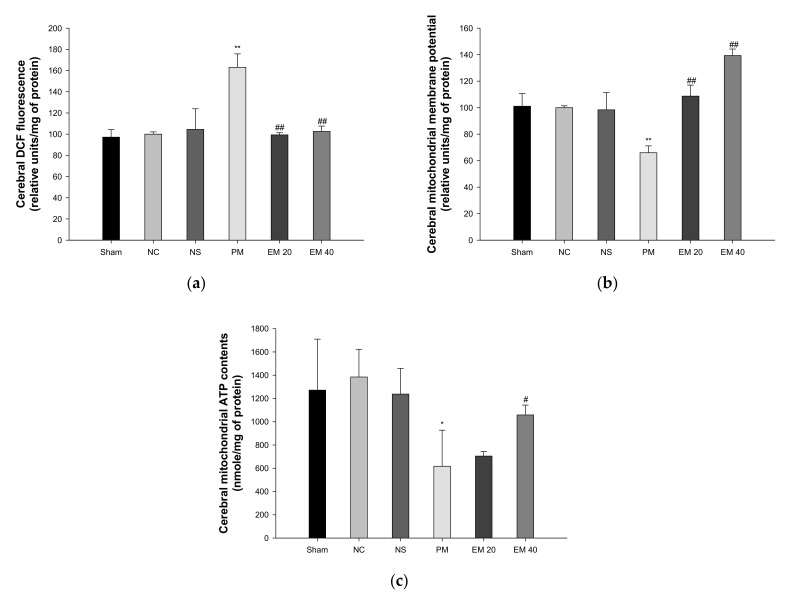
Protective effect of aqueous extract of matcha (EM) on mitochondrial DCF fluorescence (**a**), mitochondrial membrane potential (**b**), and mitochondrial ATP contents (**c**) in cerebral tissue. Results shown are mean ± SD (*n* = 5). Data were statistically considered at *p* < 0.05, and data are statistically represented with * = significantly different from the NC group, and ^#^ = significantly different from PM group; * and ^#^ *p* < 0.05, ** and ^##^ *p* < 0.01.

The mitochondrial ROS levels in the brain tissues are shown in Figure 6a. The mitochondrial ROS contents in brain tissues among the Sham (97.19%), NC (100.00%), and NS (104.50%) groups showed no significant differences. Those of the PM group (163.02%) were significantly more increased than in the NC group. However, those of the EM groups (EM20, 99.33%; EM40, 102.61%) in the brain tissues were significantly more decreased than in the PM group.

#### 3.4.2. MMP

The MMP levels in the brain tissues are shown in Figure 6b. The MMP levels in the brain tissues among the Sham (101.09%), NC (100.00%), and NS (98.36%) groups showed no significant differences. Those of the PM group (66.00%) were significantly more reduced than in the NC group. However, those of the EM groups (EM20, 108.72%; EM40, 139.35%) in the brain tissues were significantly more increased than the PM group.

#### 3.4.3. ATP Contents

The ATP levels in the brain tissues are shown in Figure 6c. The ATP contents in the lung and brain tissues among the Sham (1272.09 nmole/mg of protein), NC (1384.75 nmole/mg of protein), and NS (1237.91 nmole/mg of protein) groups showed no significant differences. Those of the PM group (616.77 nmole/mg of protein) were significantly more reduced than in the NC group. However, those of the EM groups (EM20, 704.89 nmole/mg of protein; EM40, 1058.34 nmole/mg of protein) in brain tissues were significantly more increased than in the PM group.

### 3.5. Protein Expression in Pulmonary Tissue

**Figure 7:**
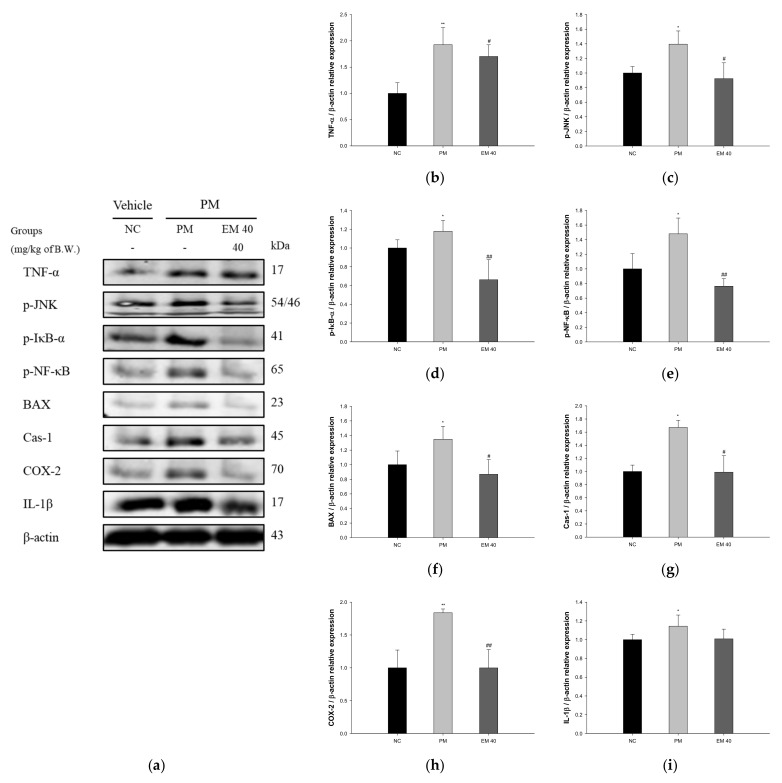
Protective effect of aqueous extract of matcha (EM) on protein expression of Western blot images (**a**). Protein expression levels of TNF- α (**b**), p-JNK (**c**), p-IκB-α (**d**), p-NF-κB (**e**), BAX (**f**), Cas-1 (**g**), COX-2 (**h**) and IL-1β (**i**) in pulmonary tissues. Results shown are mean ± SD (*n* = 3). Data were statistically considered at *p* < 0.05, and data are statistically represented with * = significantly different from the NC group, and ^#^ = significantly different from PM group; * and ^#^ *p* < 0.05, ** and ^##^ *p* < 0.01.

The pulmonary protein expressions related to inflammation and the apoptotic pathway are presented in Figure 7. TNF-α (198.75%), phosphorylated c-Jun N-terminal kinases (p-JNK) (139.54%), phosphorylated nuclear factor of kappa light polypeptide gene-enhancer in B-cells inhibitor, alpha (p-IκB-α) (117.82%), phosphorylated nuclear factor kappa-light-chain-enhancer of activated B cells (p-NF-κB) (148.01%), B-cell lymphoma 2 (BCl-2)-associated X protein (BAX) (134.84%), caspase-1 (Cas-1) (167.50%), cyclooxygenase-2 (COX-2) (183.80%), and IL-1β (114.38%) expression levels in the PM group were significantly upregulated compared to those in the NC group (100%). The EM40 group statistically downregulated TNF-α (170.33%), p-JNK (92.30%), p-IκB-α (66.18%), p-NF-κB (76.26%), BAX (86.87%), Cas-1 (98.94%), COX-2 (99.91%), and IL-1β (100.87%) expression levels compared to the PM group.

### 3.6. Protein Expression in Dermal Tissue

**Figure 8:**
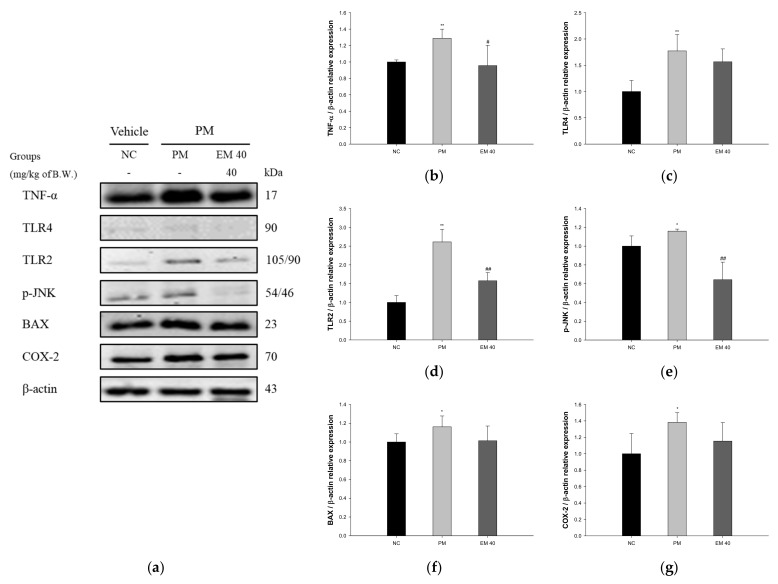
Protective effect of aqueous extract of matcha (EM) on protein expression of Western blot images (**a**). Protein expression levels of TNF-α (**b**), TLR4 (**c**), TLR2 (**d**), p-JNK (**e**), BAX (**f**), and COX-2 (**g**) in dermal tissues. Results shown are mean ± SD (*n* = 3). Data were statistically considered at *p* < 0.05, and data are statistically represented with * = significantly different from the NC group, and ^#^ = significantly different from PM group; * and ^#^ *p* < 0.05, ** and ^##^ *p* < 0.01.

The dermal protein expressions related to inflammation and the apoptotic pathway are presented in Figure 8. TNF-α (128.87%), Toll-like receptor 4 (TLR4) (177.26%), Toll-like receptor 2 (TLR2) (261.16%), p-JNK (115.99%), BAX (116.21%), and COX-2 (138.29%) expression levels in the PM group were significantly upregulated compared to those in the NC group (100%). The EM40 group statistically downregulated TNF-α (95.57%), TLR4 (156.64%), TLR2 (157.62%), p-JNK (64.23%), BAX (101.35%), and COX-2 (115.42%) expression levels compared to the PM group.

### 3.7. Protein Expression in Olfactory Bulb Tissue

**Figure 9:**
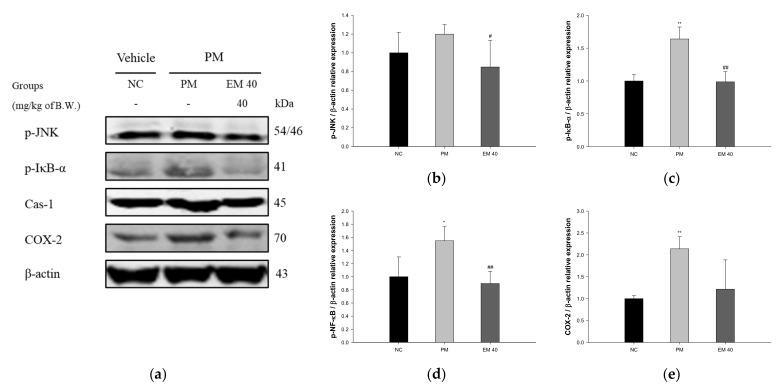
Protective effect of aqueous extract of matcha (EM) on protein expression of Western blot images (**a**). Protein expression levels of p-JNK (**b**), p-IκB-α (**c**), Cas-1 (**d**), and COX-2 (**e**) in olfactory bulb tissues. Results shown are mean ± SD (*n* = 3). Data were statistically considered at *p* < 0.05, and data are statistically represented with * = significantly different from the NC group, and ^#^ = significantly different from PM group; * and ^#^ *p* < 0.05, ** and ^##^ *p* < 0.01.

Olfactory bulb protein expressions related to inflammation are presented in Figure 9. p-JNK (119.92%), p-IκB-α (163.94%), Cas-1 (154.72%), and COX-2 (213.73%) expression levels in the PM group were significantly upregulated compared to those in the NC group. The EM40 group statistically downregulated p-JNK (84.84%), p-IκB-α (98.72%), Cas-1 (89.61%), and COX-2 (121.64%) expression levels compared to the PM group.

### 3.8. Protein Expression in Hippocampal Tissue

**Figure 10:**
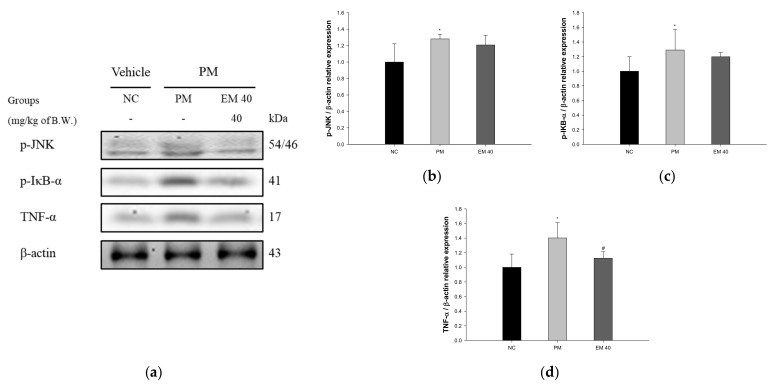
Protective effect of aqueous extract of matcha (EM) on protein expression of Western blot images (**a**). Protein expression levels of p-JNK (**b**), p-IκB-α (**c**), and TNF-α (**d**) in hippocampal tissues. Results shown are mean ± SD (*n* = 3). Data were statistically considered at *p* < 0.05, and data are statistically represented with * = significantly different from the NC group, and ^#^ = significantly different from PM group; * and ^#^ *p* < 0.05, ** and ^##^ *p* < 0.01.

Hippocampal protein expressions related to inflammation are presented in Figure 10. p-JNK (128.15%), p-IκB-α (129.03%), and TNF-α (140.21%) expression levels in the PM group were significantly upregulated compared to those in the NC group. The EM40 group statistically downregulated p-JNK (120.80.34%), p-IκB-α (119.80%), and TNF-α (112.48%) expression levels compared to the PM group.

**Figure 11:**
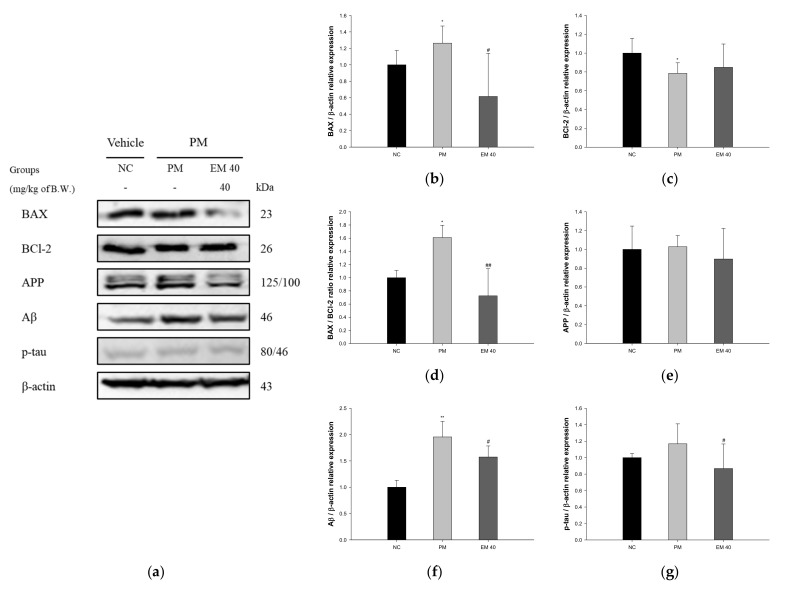
Protective effect of aqueous extract of matcha (EM) on protein expression of Western blot images (**a**). Protein expression levels of BAX (**b**), BCl-2 (**c**), BAX/BCl-2 ration (**d**), APP (**e**), Aβ (**f**), and p-tau (**g**) in hippocampal tissues. Results shown are mean ± SD (*n* = 3). Data were considered statistically significant at *p* < 0.05, and data are statistically represented with * = significantly different from the NC group, and ^#^ = significantly different from PM group; * and ^#^ *p* < 0.05, ** and ^##^ *p* < 0.01.

Hippocampal protein expressions related to neuro-cytotoxicity signaling are presented in Figure 11. BAX (126.34%), amyloid precursor protein (APP) (102.83%), amyloid β (Aβ) (195.51%), and phosphorylated tau (p-tau) (116.87%) expression levels in the PM group were significantly upregulated compared to that in the NC group. The EM40 group statistically downregulated BAX (61.55%), APP (89.72%), Aβ (157.37%), and p-tau (86.79%) expression levels compared to the PM group. BCl-2 (78.51%) expression levels in the PM group were significantly downregulated compared to that in the NC group. The EM40 group statistically upregulated BCl-2 (84.87%) expression levels compared to the PM group.

**Figure 12:**
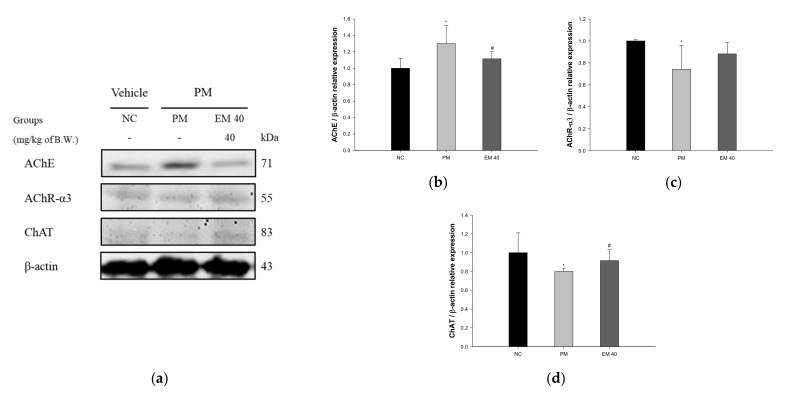
Protective effect of aqueous extract of matcha (EM) on protein expression of Western blot images (**a**). Protein expression levels of AChE (**b**), AChR-3α (**c**), and ChAT (**d**) in hippocampal tissues. Results shown are mean ± SD (*n* = 3). Data were statistically considered at *p* < 0.05, and data are statistically represented with * = significantly different from the NC group, and ^#^ = significantly different from PM group; * and ^#^ *p* < 0.05, ** and ^##^ *p* < 0.01.

Hippocampal protein expressions related to the cholinergic system are presented in Figure 12. The AChE expression levels in the PM group (130.15%) were significantly upregulated compared to those in the NC group. The EM40 group (111.57%) statistically downregulated AChE expression levels compared to the PM group. Acetylcholine receptor α3 subunits (AChR-α3) (74.18%) and choline acetyltransferase (ChAT) (80.07%) expression levels in the PM group were significantly reduced compared to those in the NC group. The EM40 group statistically upregulated AChR-α3 (88.17%) and ChAT (91.57%) expression levels compared to the PM group.

## 4. Discussion

PM is an environmental component of air pollution that leads to various health problems [2]. PM_2.5_ is not only absorbed into the body directly through the nasal cavity, lungs, and skin, but it also stimulates tissue damage, initiating an inflammatory response [26]. The absorbed PM_2.5_ and the generated cytokines pass through the BBB in the bloodstream and cause a cerebral inflammatory reaction and production of oxidative stress, leading to cognitive impairment [6]. The specific mechanism related to the systemic inflammation improvement effect of matcha is not clear. Therefore, this study was conducted to confirm the anti-amnesic effect of matcha on systemic inflammation derived from PM_2.5_ inhalation and absorption.

In general, some of PM_2.5_ is blocked by the BBB, but nano-sized particles and metal ions can enter the brain tissue through the BBB and cerebral spinal fluid, damaging the brain [1]. In particular, lead (Pb), aluminum (Al), and manganese (Mn) are toxic due to their easy migration to the hippocampus, and they increase the release of glutamate out of neuronal cells, causing glutamate excitatory neurotoxicity, neuronal apoptosis, and necrosis [27]. Therefore, PM_2.5_ enters the blood circulation after pulmonary deposition, reaches other organs, such as the brain, damages the hippocampal tissue, and results in a decrease in learning and memory function [1]. In this study, similar to previous studies, PM damaged behavior and memory functions. However, EM significantly improved PM_2.5_-induced cognitive dysfunction in mice (Figure 1 and Figure 2). In a previous study, matcha ameliorated behavioral and memory dysfunction in high-fat diet (HFD)-induced diabetic mice through the regulation of systemic inflammation [11]. Green tea contains many polyphenol compounds, and gallic acid in green tea ameliorated anxiety, depression, and locomotion behaviors by inhibiting changes in the BBB and hippocampal structure in rats [28]. Green tea polyphenol, including the various catechins, attenuated isoflurane-induced long-term learning and memory deficits via modulating oxidative stress [29]. Based on these results, green tea showed cognitive function improvement in various animal models, and EM also has a considerable anti-amnesic effect derived from numerous physiological polyphenols against PM_2.5_-induced cognitive deficits.

Exposure to PM_2.5_ causes oxidative stresses throughout the body, causing an imbalance in the antioxidant systems by reducing the contents of CAT, GSH, and SOD, and increasing the lipid peroxidation in a variety of organs, such as the brain, liver, lungs, colon, and kidneys [30]. In particular, lung and skin tissues have a structure that is vulnerable to fine dust due to direct damage from PM_2.5_, and damages in lung and skin tissues cause an inflammatory reaction by generating cytokines throughout the whole body [31]. In addition, PM_2.5_ easily penetrates brain tissue and causes an inflammatory response of neuronal cells through the stimulation of microglial cells [32]. The systemic inflammatory response increases the production of free radicals, such as hydroxyl radicals and superoxide. Oxidative stress generated by this process ultimately causes the reduction of antioxidant enzymes in organs as well as brain damage, impairing cognitive function [33]. To evaluate the ameliorating effect of EM on antioxidant system damage, reduced GSH levels and MDA production in pulmonary, dermal, and cerebral tissues were assessed (Figure 4). Based on previous studies, PM damages the antioxidant system in lung, skin, and brain tissues. However, EM has shown a considerable protective effect of the antioxidant system in PM_2.5_-exposed tissues. Similar to this study, green tea protected against hepatic antioxidant dysfunction by reducing benzo(a)pyren, one of the PAHs, as well as against induced oxidative stress and DNA damage [34]. L-theanine attenuated lipid peroxide and ROS production and elevated the GSH and catalase levels in cadmium (Cd)-exposed mice brains [35]. In addition, tea-derived polyphenols inhibited pulmonary apoptosis via the regulation of MDA contents and SOD activity in PM_2.5_-exposed pulmonary A549 cells [36]. In conclusion, the consumption of EM effectively protected the antioxidant system in pulmonary, dermal, and cerebral tissues against PM_2.5_ toxicity. It is judged to have a protective effect against PM_2.5_ exposure and to prevent cognitive impairment by protecting the antioxidant system of brain tissue.

Various heavy metals contained within fine dust have a negative effect on the function of the cholinergic system. In particular, damage to the cholinergic system is closely related to the development of AD and Parkinson’s disease [37]. In general, the cholinergic system regulates the neurotransmission system in brain tissue and regulates the physiological functions of memory and learning. However, heavy metals, oxidizing agents, and sulfur in fine dust binds to the cholinergic system, forming a complex that causes changes in the protein structure, changes in the enzymatic system, and cell dysfunction [38]. This ultimately inhibits transmission between neurons, resulting in neuronal cell death and cognitive impairment [37]. However, the administration of EM ameliorated cholinergic dysfunction by regulating the ACh contents and AChE activity in brain tissue, especially hippocampal tissue (Figure 5 and Figure 12). Green tea catechins protect against lead and Cd-induced ischemia by regulating the cholinergic system [39]. Matcha restored the cholinergic system by regulating the ACh contents and expressions of AChE and ChAT in mice brain tissues of HFD-induced diabetic cognitive dysfunction [11]. In addition, green tea catechins, including EC, ECG, EGC, and EGCG, statistically inhibited the activity of AChE and butyryl cholinesterase [40]. The cholinergic system protective effect of EM appears to be due to various catechins and physiologically active substances contained within EM. In addition, because EM contains higher catechin contents than leaf green tea, physiological activities might be more considerable than green tea (Table S1) [8]. The improvement of the cholinergic system through the ingestion of EM might ultimately help improve cognitive function.

PM_2.5_ induces mitochondrial dysfunction by causing the production of oxidative stress in cells and inhibiting energy supply [41]. In particular, PM_2.5_ can directly enter the cytoplasm of cells, and many vacuoles and swollen mitochondria were observed in the cytoplasm, and varisized vacuoles and swollen mitochondria were found [42]. Exposure to PM_2.5_ induces cell cycle arrest in the G0/G1 stage and DNA damage, activating the death signaling pathway. It could also damage the mitochondrial structure and promote energy metabolism [43]. Moreover, PM_2.5_ causes mitochondrial damage, including mitochondrial membrane rupture, cristae fragmentation, and lysis, and induces the accumulation of free radicals through the disruption of antioxidant defense systems, leading to apoptosis and neuronal dysfunction [39], whereas the intake of EM protected against mitochondrial dysfunction by attenuating the production of ROS, MMP deficit, and reduced ATP contents (Figure 6). Similar to this research, a variety of physiologically active compounds in green tea were found to have an improvement effect on mitochondrial damage. EGCG protected against Cd-induced mitochondrial dysfunction by reducing lipid peroxide and decreasing GSH contents [44]. Chlorogenic acid promotes the excretion of Al from hippocampal mitochondria through chelation and inhibits the depolarization and reduction of creatine kinase activity, thereby decreasing the rate of ATP hydrolysis [45]. In addition, L-theanine, one of the amino acids in green tea, prevented cadmium chloride-induced disruption of transmembrane potential from mitochondrial damage by modulating the BAX/BCl-2 ratio as well as cleaved caspase-9, caspase-3, and poly (ADP-ribose) polymerases in pheochromocytoma PC12 cells [46]. In conclusion, the large amounts of physiologically active compounds in EM inhibit mitochondrial damage caused by the toxicity of various heavy metals present in fine dust. Thus, EM is ultimately judged to be a material that can help improve cognitive function.

Lung tissue is easily damaged by exposure to PM_2.5_, which stimulates the inflammatory response of the nasal cavity and respiratory tract and increases inflammatory cytokines, such as interleukin-2 (IL-2), interleukin-12 (IL-12), interleukin-17A (IL-17A), interferon gamma (IFNγ), monocyte chemoattractant protein-1(MCP-1), and soluble CD40 ligand (sCD40 L) [47]. The prolonged inflammatory response causes an increase in neutrophils, T cells, and eosinophils, which migrate to the lungs and other tissues [48]. The inflammatory reaction inside the lungs induces the release of more cytokines and chemokines, and damage to lung cells occurs due to the chronic inflammatory reaction [49]. In addition, exposure to PM_2.5_ promotes the generation of oxidative stresses, such as ROS and free radicals, and causes cell damage through a decrease in the cellular antioxidant system [25]. Exposure to PM_2.5_ induces the peroxidation of lipids in the cell membrane and increases intracellular Ca^2+^, stimulating the expression of BAX and caspases and inducing an apoptosis cascade [50]. PM_2.5_-induced inflammatory responses and the production of oxidative stress in lung tissue are systemically transmitted, and protein expressions of p-JNK, p-NF-κB, and COX-2 related to inflammation are stimulated [51]. In particular, PM_2.5_ itself is absorbed into the pulmonary blood vessels. It has been reported that absorbed PM_2.5_ and inflammatory cytokines, such as TNF-α and IL-1β, affect a variety of organs, especially brain tissue [37]. Similar to previous studies, to evaluate the ameliorating effect of EM on pulmonary inflammation, protein expression related to inflammation was assessed (Figure 7). EM significantly suppressed the expression of TNF-α, p-JNK, p-IκB-α, p-NF-κB, and COX-2, related to inflammation, and BAX and Cas-1, related to apoptosis in pulmonary tissue. Tea-derived polyphenols showed an improvement effect by regulating the protein expressions of caspase-3, BAX/BCl-2, and C/EBP-homologous protein (CHOP) and inhibiting intracellular ROS production in PM_2.5_-induced A549 cells [36]. In addition, quercetin, one of the green tea flavonoids, alleviated PM_2.5_-induced ROS generation by attenuating the mitochondrial structure and function damage caused by PM_2.5_ in human bronchial epithelial 16HBE cells [52]. Similar to previous studies, EM containing diverse polyphenol compounds effectively improved pulmonary inflammation and damage. Thus, it is considered that EM plays an important role in regulating pulmonary inflammation and apoptosis.

The skin is an organ directly affected by PM along with the nasal and respiratory tissues [53]. PM_2.5_ activates Toll-like receptor (TLR) signals that increase the expression of inflammatory cytokines, activating innate and adaptive immunity associated with Th2 responses [54]. This increases the number of neutrophils and promotes inflammation by increasing the level of cytokines, such as TNF-α and IL-1β in the dermal tissue [55]. An increased expression of TLRs by PM_2.5_, including transition metals and organic compounds, promotes the activation of extracellular signal-regulated kinase (ERK), JNK, p38 mitogen-activated protein kinase (MAPK) signals, and the NF-ҡB pathway, which reduces the phosphorylation of protein kinase B (Akt). Through this process, the ratio of BAX/BCl-2 and the expressions of caspase cascade, which induces apoptosis, increases [56]. In addition, the stimulated protein expression of the NF-ҡB pathway inhibited the expression of antioxidant enzymes, such as catalase, SOD, and heme oxygenase-1 (HO-1), by upregulating COX-2 expression [53]. In this study, EM significantly attenuated the PM_2.5_-induced inflammatory effect related to the TLR pathway by regulating the expression of TLR4 and TLR2 in dermal tissue. Caffeic acid phenethyl ester reduced the inflammatory effect by attenuating TLR4, myeloid differentiation factor 88 (MyD88), NF-κB activation, and inducible nitric oxide synthase (iNOS) in LPS-induced gingival fibroblasts cells [57]. Quercetin, which is a green tea flavonoid, inhibited the expression of TLR2 and TLR4 and nuclear translocation of the NF-κB p65 subunit in atherosclerosis rats [58]. Treatment with EGCG inhibited the increased protein expressions of NADPH oxidase-1 (NOX-1), NADPH oxidase-2 (NOX-2), TNF-α, IL-1β, IL-6, IL-8, and matrix metalloproteinase-1 (MMP-1) against PM_10_-induced cytotoxicity in human epidermal keratinocytes [59]. In addition, EGCG protected against PM_10_-induced mitochondrial dysfunction via the regulation of NF-κB, activator protein 1 (AP-1), and MAPKs expression in human dermal fibroblast cells [60]. In conclusion, EM that contains a variety of physiologic compounds, such as caffeic acid, quercetin, and catechins, significantly inhibits dermal inflammatory responses caused by PM_2.5_ toxicity by suppressing the TLR signaling pathway, and it might ultimately suppress systemic inflammation by inhibiting the secretion of inflammatory cytokines in skin tissues.

It has been reported that PM_2.5_ is directly absorbed through the nasal cavity and damages the brain. In particular, the olfactory bulb, which is closely related to the sense of smell, induces the production of macrophage inflammatory protein-1α (MIP-1α) by exposure to PM_2.5_ and promotes inflammatory responses [61]. Because the inflammatory responses of the olfactory bulb occur much earlier than in the cortical or hippocampal region of the brain, neuronal damage in the olfactory bulb has been reported as the first sign of CNS dysfunction [1]. PM_2.5_ absorbed in the nasal cavity reduces the expression of nuclear factor erythroid-2-related factor 2 (Nrf2), HO-1, and NAD(P)H quinone oxidoreductase 1 (NQO-1), increasing the levels of p-NF-κB and p-IκB-α and the levels of cytokines, such as TNF-α and IL-1β in the olfactory bulb [62]. The persistent inflammatory response in the olfactory bulb affects the whole brain, particularly the hippocampus, amygdala, and cerebral cortex [52]. Similar to these results, exposure to PM_2.5_ promoted an inflammatory response and increased the expression of p-JNK, p-IκB-α, Cas-1, and COX-2. However, EM significantly suppressed the protein expression of inflammation in the olfactory bulb (Figure 9). The administration of white tea, a kind of green tea product, improved the olfactory function of mice in the olfactory disorder model caused by chronic unpredictable mild stress of morphological and molecular levels by protecting against mitochondrial structural damage, such as cristae fracture and vacuolar degeneration [63]. In addition, the administration of chlorogenic acid and gallic acid in green tea improved the behavior dysfunction of rats subjected to bilateral olfactory bulbectomy [64]. Similar to these results, EM, which contains a large amount of phenolic acid, such as chlorogenic acid and gallic acid, might help protect cognitive function by inhibiting damage to the olfactory bulb, which causes early-stage cognitive dysfunction.

Recently, studies on the association between air pollution and AD, which is a type of senile dementia and is accompanied by cognitive impairment, are ongoing, and exposure to environmental toxicity has been reported to cause neuroinflammation and neuropathology [65]. PM_2.5_ exposure increases ROS levels in neurons and increases the expression of oligomer Aβ. In addition to oligomer Aβ, it promotes ROS production by the activation of NADPH oxidase and mitochondrial damage and stimulates the secretion of cytokines, such as IL-1β, through the activation of cryopyrin inflammasome in microglia [6]. Furthermore, it plays a major role in causing neuronal cell apoptosis and synaptic damage by inhibiting p-ERK1/2 and p-CREB [66]. Exposure to PM_2.5_ promotes apoptosis and the production of Aβ, as well as various inflammatory reactions, leading to cognitive impairment. Based on previous studies, to evaluate the ameliorating effect of EM on hippocampal damage, protein expression related to the Aβ/tau pathway was assessed (Figure 10, Figure 11 and Figure 12). PM_2.5_ stimulated inflammatory proteins, such as p-JNK, p-IκB-α, and TNF-α, apoptosis signaling, such as the BAX/BCl-2 ratio, and cytotoxicity proteins related to cognitive function, such as Aβ and p-tau, and EM significantly ameliorated hippocampal dysfunction. In a previous study, EM attenuated the protein expression of p-JNK, p-tau, and Aβ by regulating the p-Akt, brain-derived neurotrophic factor (BDNF) and insulin-degrading enzyme (IDE) expression related to the JNK pathway in HFD-induced diabetic mice [11]. In addition, the administration of L-theanine in green tea attenuated the phosphorylation of tau protein by downregulating the phosphorylation of glycogen synthase kinase 3 beta (GSK-3β) (Ser 9), mammalian target of rapamycin (mTOR) (Ser 2448), and ribosomal protein S6 kinase beta-1 (S6K1) (Thr 389) in Cd-induced neurotoxicity [35]. Al, which is in PM, increases apoptotic and amyloidogenic cell damage in the hippocampus. However, black tea extract, a green tea product, significantly suppressed the release of cytochrome c into cytosol and the protein expression of BAX, caspase-3, caspase-9, and caspase-8, and downregulated the expression of APP and Aβ_1-42_ by reducing β-secretase and γ-secretase related to the accumulation of amyloid plaque [67]. EM inhibited cerebral neuronal death by regulating the expression of hippocampus inflammatory factors and various proteins. Ultimately, it is suggested that EM might be a material that can help improve cognitive function by regulating the systemic inflammatory response in a variety of tissues, including the hippocampus. Therefore, through in vivo experiments, EM is expected to be used as a material for functional food that effectively eliminates the toxicity of PM_2.5_.

## 5. Conclusions

Research on the toxicity caused by air pollution continues. However, targeting therapeutics is difficult because the pathways of PM_2.5_ toxicity are not precise. In addition, as a drug to treat fine dust has not been developed, the risk continues to increase. Therefore, it is very important to prevent cognitive dysfunction caused by fine dust toxicity before it appears. Thus, this study evaluated that powdered green tea (matcha) inhibited cognitive dysfunction and PM_2.5_-induced neuronal cytotoxicity by regulating systemic inflammation in BALB/c mice. EM ameliorated behavioral and memory dysfunctions, and it promoted pulmonary, dermal, and cerebral antioxidant systems. Moreover, EM improved the cerebral cholinergic system by regulating cerebral ACh contents, AChE activity, and mitochondrial functions. It attenuated the expression of PM_2.5_-induced proteins involved in inflammation, such as TNF-α, p-JNK, p-IκB-α, p-NF-κB, BAX, Cas-1, COX-2, and IL-1β in lung tissue, and TNF-α, TLR4, TLR2, p-JNK, BAX, and COX-2 in dermal tissue. EM also regulated the expression of p-JNK, p-IκB-α, Cas-1, and COX-2 in olfactory bulb tissues, and p-JNK, p-IκB-α, TNF- α, BAX, BCl-2, APP, Aβ, and p-tau in hippocampus tissues. In addition, EM protected the cholinergic system in hippocampal tissue by regulating the expression of AChE, AChR-α3, and ChAT. These findings suggest that EM is a potential functional food material to improve PM_2.5_-induced cognitive dysfunctions by regulating systemic inflammation in various tissues.

## Figures and Tables

**Table 1:** Inorganic components of PM_2.5_ (Unit: mg/g).

Al	Fe	Mg	Mn	Ba	Zn	Cu	Pb	Li	Cr	Co	Cd
30.36 ± 6.41	16.91 ± 3.12	6.26 ± 1.20	0.58 ± 0.14	0.22 ± 0.06	0.07 ± 0.02	0.05 ± 0.01	0.03 ± 0.01	0.02 ± 0.01	0.01 ± 0.00	0.00 ± 0.00	0.00 ± 0.00

Results shown as mean ± SD (*n* = 3).

**Table 2:** List of primary antibodies and their information used in this study.

Antibody	Catalog	Concentration	Manufacturer
β-actin	sc-69879	1:1000	Santa Cruz Biotech. (Dallas, TX, USA)
p-JNK	sc-6254	1:1000	Santa Cruz Biotech. (Dallas, TX, USA)
p-IκB-α	sc-8404	1:1000	Santa Cruz Biotech. (Dallas, TX, USA)
BAX	sc-7480	1:1000	Santa Cruz Biotech. (Dallas, TX, USA)
Caspase-1	sc-392736	1:1000	Santa Cruz Biotech. (Dallas, TX, USA)
COX-2	sc-376861	1:1000	Santa Cruz Biotech. (Dallas, TX, USA)
iNOS	sc-7271	1:1000	Santa Cruz Biotech. (Dallas, TX, USA)
APP/Aβ	sc-28365	1:1000	Santa Cruz Biotech. (Dallas, TX, USA)
p-tau	sc-12952	1:1000	Santa Cruz Biotech. (Dallas, TX, USA)
AChE	sc-373901	1:1000	Santa Cruz Biotech. (Dallas, TX, USA)
TLR2	sc-21759	1:1000	Santa Cruz Biotech. (Dallas, TX, USA)
TLR4	sc-52962	1:1000	Santa Cruz Biotech. (Dallas, TX, USA)
BCl-2	sc-509	1:1000	Santa Cruz Biotech. (Dallas, TX, USA)
AChR-α3	sc-365479	1:1000	Santa Cruz Biotech. (Dallas, TX, USA)
TNF-α	5178SC	1:1000	Cell Signaling Tech. (Danvers, MA, USA)
ChAT	20747-1AP	1:1000	Bioneer (Daejeon, Korea)

## Data Availability

The data underlying this article are shared upon reasonable request to the corresponding author.

## Supplementary Materials

The following are available online at https://www.mdpi.com/article/10.3390/antiox10121932/s1, Table S1: Catechin contents of aqueous extract of leaf green tea and matcha green tea, Table S2: Antioxidant capacity of aqueous extract of leaf green tea and matcha green tea.
