# Supplementary material for: Powdered Green Tea (Matcha) Attenuates the Cognitive Dysfunction via the Regulation of Systemic Inflammation in Chronic PM2.5-Exposed BALB/c Mice
Source: Antioxidants (Basel). 2021 Nov 30;10(12):1932. doi: 10.3390/antiox10121932 (PMC8750520; doi:10.3390/antiox10121932)
Supplement: Supplementary file 1 [file antioxidants-10-01932-s001.zip › antioxidants-1469993-supplementary.pdf]

Table S1. Catechin contents of aqueous extract of leaf green tea and matcha green tea. [1)]

(Unit: mg/g)

	**EGC**	**EC**	**EGCG**	**ECG**	**Total catechin**
Leaf green tea	39.27±0.58[b]	5.22±0.21[b]	38.18±0.08[b]	10.57±0.07[b]	93.24±0.33[b]
Matcha green tea	49.49±0.12[a]	8.74±0.06[a]	50.24±0.57[a]	13.75±0.11[a]	122.22±0.21[a]

Results are mean±SD (n=3). Data were statistically represented at p<0.05, and different small alphabets mean statistical significance.

Table S2. Antioxidant capacity of aqueous extract of leaf green tea and matcha green tea. [1)]

	**TPC[1]**	**TFC[2]**	**ABTS[3]**	**DPPH[4]**	**MDA[5]**
Leaf green tea	235.92±1.25[b]	83.87±1.03[b]	310.48±2.48[a]	380.98±5.15[a]	68.15±3.57[a]
Matcha green tea	325.00±7.35[a]	104.24±5.17[a]	276.56±4.25[b]	321.05±3.24[b]	59.98±2.48[b]

[1]TPC, total phenolic content; [2]TFC, total flavonoid content; [3]ABTS, ABTS radical scavenging activity; [4]DPPH, DPPH radical scavenging activity; [5]MDA, malondialdehyde (MDA) inhibitory effect. Results shown are mean±SD (n=3). Data were statistically represented at p<0.05, and different small alphabets mean statistical significance. Results of TPC and TFC are presented as mg of GAE/g and mg of RE/g, respectively. Results of ABTS, DPPH and MDA are presented as IC_{50} value (μg/mL).

[1)] Kim, J. M. et al., (2021). Protective effect of matcha green tea (*Camellia sinensis*) extract on high glucose-and oleic acid-induced hepatic inflammatory effect. Korean Journal of Food Science and Technology, 53(3), 267-277.
